# Hypoperfusion of brain parenchyma is associated with the severity of chronic cerebrospinal venous insufficiency in patients with multiple sclerosis: a cross-sectional preliminary report

**DOI:** 10.1186/1741-7015-9-22

**Published:** 2011-03-07

**Authors:** Paolo Zamboni, Erica Menegatti, Bianca Weinstock-Guttman, Michael G Dwyer, Claudiu V Schirda, Anna M Malagoni, David Hojnacki, Cheryl Kennedy, Ellen Carl, Niels Bergsland, Christopher Magnano, Ilaria Bartolomei, Fabrizio Salvi, Robert Zivadinov

**Affiliations:** 1 Vascular Diseases Center, University of Ferrara-Bellaria Neurosciences, Ferrara and Bologna, Italy; 2 The Jacobs Neurological Institute, University at Buffalo, Buffalo, NY, USA; 3 Buffalo Neuroimaging Analysis Center, University at Buffalo, Buffalo, NY, USA

## Abstract

**Background:**

Several studies have reported hypoperfusion of the brain parenchyma in multiple sclerosis (MS) patients. We hypothesized a possible relationship between abnormal perfusion in MS and hampered venous outflow at the extracranial level, a condition possibly associated with MS and known as chronic cerebrospinal venous insufficiency (CCSVI).

**Methods:**

We investigated the relationship between CCSVI and cerebral perfusion in 16 CCSVI MS patients and 8 age- and sex-matched healthy controls. Subjects were scanned in a 3-T scanner using dynamic susceptibility, contrast-enhanced, perfusion-weighted imaging. Cerebral blood flow (CBF), cerebral blood volume (CBV) and mean transit time (MTT) were measured in the gray matter (GM), white matter (WM) and the subcortical GM (SGM). The severity of CCSVI was assessed according to the venous hemodynamic insufficiency severity score (VHISS) on the basis of the number of venous segments exhibiting flow abnormalities.

**Results:**

There was a significant association between increased VHISS and decreased CBF in the majority of examined regions of the brain parenchyma in MS patients. The most robust correlations were observed for GM and WM (*r *= -0.70 to -0.71, *P *< 0.002 and *P *corrected = 0.022), and for the putamen, thalamus, pulvinar nucleus of thalamus, globus pallidus and hippocampus (*r *= -0.59 to -0.71, *P *< 0.01 and *P *corrected < 0.05). No results for correlation between VHISS and CBV or MTT survived multiple comparison correction.

**Conclusions:**

This pilot study is the first to report a significant relationship between the severity of CCSVI and hypoperfusion in the brain parenchyma. These preliminary findings should be confirmed in a larger cohort of MS patients to ensure that they generalize to the MS population as a whole. Reduced perfusion could contribute to the known mechanisms of virtual hypoxia in degenerated axons.

## Background

Chronic cerebrospinal venous insufficiency (CCSVI) is a vascular condition described in multiple sclerosis (MS) patients and characterized by multiple intraluminal stenosing malformations of the principal pathways of extracranial venous drainage, particularly in the internal jugular veins (IJVs) and the azygous vein (AZY), that restrict the normal outflow of blood from the brain [1,2].

The concept of CCSVI in MS patients and its possible implications on MS pathogenesis and treatment options has raised significant interest in both the patient and medical communities. Surgical interventions are under consideration in patients with MS and it is important to understand the relevance of CCSVI in the context of well-established hemodynamic brain MRI outcomes. Several studies have reported hypoperfusion of the brain parenchyma in MS patients and also preceding disease onset [3,4]. Changes in perfusion MRI parameters are relevant in MS pathogenesis because they represent the necessary step inducing a status defined as a hypoxia-like condition [5].

It is possible that the presence and severity of venous outflow blockages characterizing CCSVI may contribute to reduced cerebral perfusion. In this study, we investigated whether impaired venous outflow is related to hypoperfusion of brain parenchyma.

## Methods

Sixteen consecutive relapsing-remitting (RR) MS patients and eight age- and sex-matched healthy controls (HC) were enrolled in this study as previously described [6]. Briefly, the inclusion criteria required clinically definitive MS, RR MS disease course, an Expanded Disability Status Scale (EDSS) score between 0 and 5.5, age 18 to 65 years, disease duration between 5 and 10 years, being treated with currently U.S. Food and Drug Administration-approved, disease-modifying treatments and having normal renal function (creatinine clearance >58 ml/min). Exclusion criteria included an acute relapse and/or steroid treatment within the 30 days preceding study entry, preexisting medical conditions associated with brain pathology and abnormal renal function.

All investigators conducting assessments were blinded to the clinical, demographic, and subject group (MS or HC) characteristics. We aimed to ensure proper blinding by instructing subjects not to reveal their disease status during the Doppler examination and including RR MS patients with low disability or walking difficulties. The Italian research group conducted the Doppler assessment, and the Buffalo research group conducted clinical and magnetic resonance imaging (MRI) examinations. The clinical, Doppler and MRI assessments were conducted on the same day for each subject.

The study was approved by the institutional review board, and written informed consent was obtained from all study subjects.

### MRI scan acquisition and analysis

All subjects were examined on a 3-T GE Signa Excite scanner (General Electric, Milwaukee, WI, USA). The following sequences were acquired: two-dimensional (2-D) multiplanar dual fast spin-echo proton density and T2-weighted images, fluid-attenuated inversion recovery (FLAIR) images, a 3-D high-resolution (HIRES) fast spoiled gradient echo (FSPGR) with magnetization-prepared inversion recovery (IR) pulse- and perfusion-weighted imaging (PWI).

One average was used for all pulse sequences. With the exception of PWI, all sequences were acquired with a 256 × 192 matrix (frequency × phase) and field of view (FOV) of 25.6 cm × 19.2 cm (256 × 256 matrix with phase FOV = 0.75) for an in-plane resolution of 1 mm × 1 mm. For all 2-D scans (PD/T2, FLAIR and SE T1), 64 slices were collected with a thickness of 2 mm and no gap between slices. For the 3-D HIRES IR-FSPGR, 184 locations were acquired, 1 mm thick, providing for isotropic resolution. Other relevant parameters were as follows. For dual FSE PD/T2, echo and repetition times (TE and TR) TE_1_/TE_2_/TR = 9/98/5300 ms; flip angle (FA) = 90; echo train length (ETL) = 14; and acquisition time (AT) = 5:08 (min:sec). For FLAIR images, TE/TI/TR = 120/2100/8500 ms (TI inversion time), FA = 90, ETL = 24 and AT = 6:49. For SE T1-WI, TE/TR = 16/600 ms, FA = 90 and AT = 6:11. For 3-D HIRES T1-WI, TE/TI/TR = 2.8/900/5.9 ms, FA = 10 and AT = 9:18.

Dynamic susceptibility contrast-enhanced PWIs were acquired during and after injection of 15 ml of 0.1 mM/kg gadolinium-diethylenetriamine penta-acetic acid with an MRI-compatible power injector at a speed of 5 ml/s. The HC were also injected with the contrast agent. Single-shot, gradient-echo, echo planar imaging was used with the following parameters: TR 2275 ms, TE 45 ms, FOV 26 × 26 cm, matrix 96 × 96 (resulting in in-plane voxel sizes of 2.71 × 2.71 mm), 20 slices (7 mm thick) with no gap. Forty time points were acquired per slice.

PWI characteristics of the gray matter (GM) and white matter (WM) tissue compartments were assessed by using SIENAX [7]. Subcortical gray matter (SGM) structures were assessed by using FMRIB's FIRST software to segment high-resolution, 3-D, T1-weighted images http://www.fmrib.ox.ac.uk/fsl/first/index.html and included the thalamus, pulvinar nucleus of thalamus, caudate, putamen, globus pallidus, hippocampus, amygdala, nucleus accumbens, red nucleus and substantia nigra. Briefly, FIRST is a model-based segmentation/registration program that uses shape/appearance models constructed from manually segmented images. The manual labels are parameterized as surface meshes and modeled as a point distribution model. Deformable surfaces are used to automatically parameterize the volumetric labels in terms of meshes; the deformable surfaces are constrained to preserve vertex correspondence across the training data. Normalized intensities along the surface normals are sampled and modeled. The shape and appearance model is based on multivariate Gaussian assumptions. Shape is then expressed as a mean with modes of variation (principal components). On the basis of the learned models, FIRST searches through linear combinations of shape modes of variation for the most probable shape instance, given the observed intensities in the input image.

**Figure 1:**
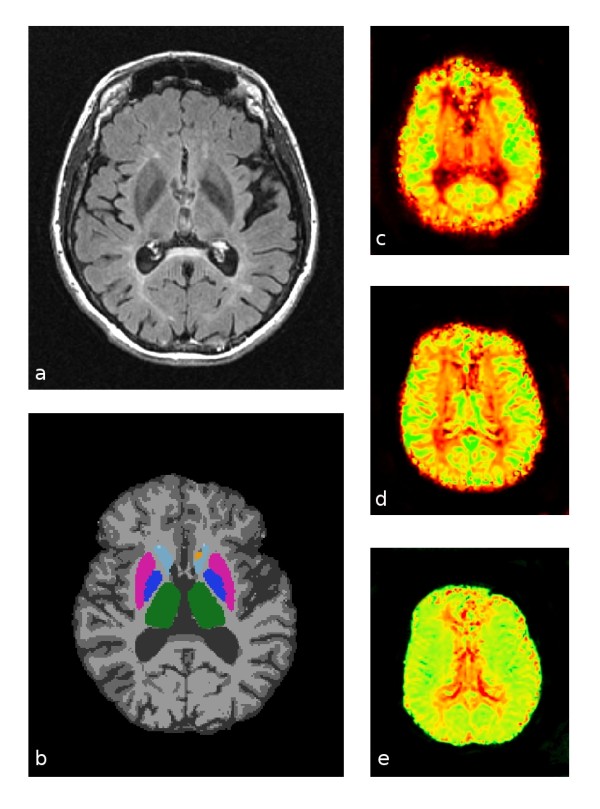
**Representative source and processed images used for perfusion calculations**. **(a) **Original fluid-attenuated inversion recovery images. **(b) **Gray, white and deep gray structure segmentations: gray matter in medium gray, white matter in light gray, thalamus in green, globus pallidus in dark blue, putamen in magenta, caudate in light blue and nucleus accumbens in orange. **(c) **Cerebral blood flow (CBF) map: low flow in red and high flow in green. **(d) **Cerebral blood volume map: low volume in red and high volume in green. **(e) **Mean transit time map: short transit time in green and long transit time in red.

Calculation of perfusion cerebral blood flow (CBF), cerebral blood volume (CBV) and mean transit time (MTT) was conducted by blinded operators using a previously described method [8]. Briefly, we used the Java Image Manipulation software package (Xinapse Systems, Thorpe Waterville, UK) with an automated additive interval-finding algorithm (searching 500 "artery-like" candidate voxels and retaining the 40 best fitting voxels) and singular value decomposition (cutoff at 20% of maximum singular value) for perfusion curve fitting [9]. CBF and CBV values were relative, based on estimated tissue relaxivity and hematocrit parameters (arterial relaxivity 1.0 L/s/M, tissue relaxivity 1.0 L/s/M, arterial hematocrit 0.45, tissue hematocrit 0.45). Correction for patient motion prior to perfusion analysis was performed using FMRIB's Linear Image Registration Tool for Motion Correction (MCFLIRT). Using the first steady-state volume before contrast arrival time, we applied FMRIB's Linear Image Registration Tool (FLIRT) to derive an affine transformation matrix, providing a transformation from the native perfusion acquisition space to the high-resolution FLAIR space. This matrix was then used to coregister perfusion MTT, CBF and CBV maps into the subject-specific upsampled FLAIR space. These maps were then overlaid onto all relevant region-of-interest masks to calculate mean values for MTT, CBF and CBV within each tissue compartment (Figure 1).

### Assessments of cerebral venous hemodynamics

Cerebral venous return was examined using echo-color Doppler equipped with 2.5 and 7.5 to 10 MHz transducers (Esaote-Biosound My Lab 25, Genoa, Italy), with the subject positioned on a bed tilted at 90° and 0° as previously described [2].

**Figure 2:**
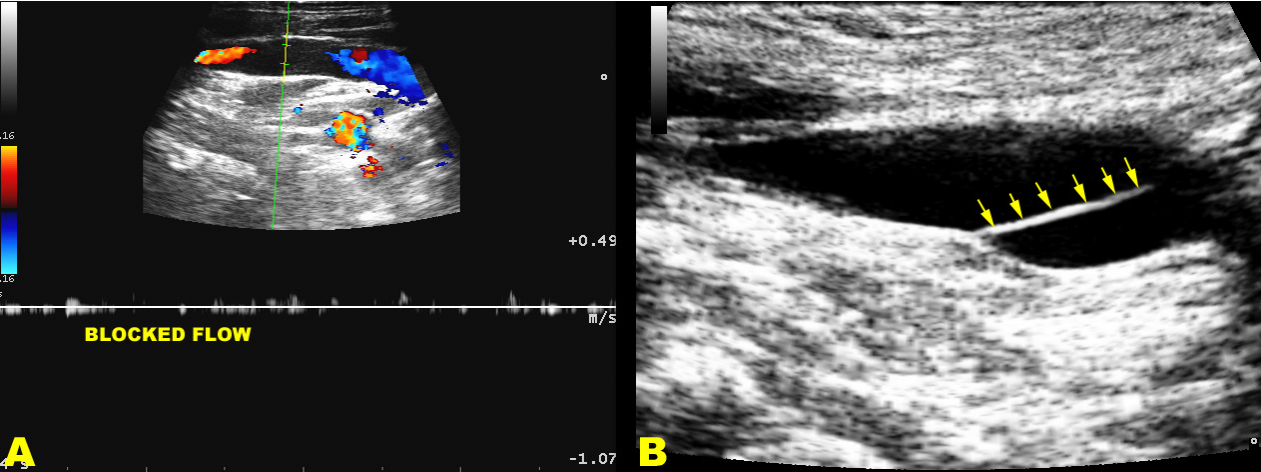
**Ultrasound assessment in CCSVI**. **(a)** Triplex scanner, longitudinal access of the neck in chronic cerebrospinal venous insufficiency multiple sclerosis patient. In the distal internal jugular vein, close to the junction, the flow is blocked as demonstrated both by the absence of color and by the Doppler spectrum analysis, with the sample completely open in the lumen and no angle correction. **(b) **An immobile intraluminal defect of the defined septum (multiple arrows) almost completely obstructing the lumen shows the cause of the hampered venous outflow.

All subjects were scanned in a blinded manner following the established protocol for diagnosis of CCSVI [2], consisting of transcranial and extracranial echo-color Doppler to measure the following five venous hemodynamic (VH) parameters indicative of CCSVI: (1) reflux in the IJVs and/or in the vertebral veins (VVs) in sitting and in supine positions (90° and 0°), with reflux defined as flow directed toward the brain for a duration of >0.88 seconds; (2) reflux in the intracranial veins with reflux defined as reverse flow for a duration of 0.5 seconds in one of the insonated veins (superior and inferior petrosus sinus, and/or Rosenthal vein); (3) B-mode abnormalities causing absence of flow or significant flow disturbances (vestigial valves, septum, immobile valve leaflets, see Figure 2), or stenoses in IJVs. IJV stenosis was defined as a cross-sectional area of this vein ≤0.3 cm^2 ^; (4) flow that is not Doppler-detectable in IJVs and/or VVs despite multiple deep breaths; and (5) a wider cross-sectional area of the IJVs in the upright positions respect to supine. A subject was considered CCSVI-positive if at least two VH criteria were fulfilled as previously proposed [2].

We also calculated a VH insufficiency severity score (VHISS), as previously described [6]. The VHISS is an ordinal measure of the overall extent and number of VH flow pattern anomalies, with a higher value of VHISS indicating a greater severity of VH flow pattern anomalies. For each of the five VH criteria, a "VHISS contribution score" was assigned using the scheme described below. These scores combined gave an overall severity measure: the VHISS. The minimum possible VHISS value is 0 and the maximum is 16.

As regards criterion VH1, there are eight venous segments that can potentially exhibit reflux in the two postures, and one point was assigned for each one at which reflux was found to be present. Consequently, VH1 had a VHISS contribution score that could range from a minimum of 0 to a maximum of 8.

Criterion VH2 was assigned a VHISS contribution score of 1 if reflux was present in the intracranial veins in only one posture and a VHISS contribution score of 2 if it was present in both postures. The VHISS contribution score for this criterion was additionally weighted with a factor of 2 if reflux toward the subcortical GM could be detected. Consequently, the VHISS contribution score for VH2 could range from a minimum of 0 to a maximum of 4.

The VHISS contribution score for VH3 ranged from 0 to 2, depending on whether B-mode anomalies disturbing outflow were present in none, one or both of the IJVs, respectively (Figure 1). VH3 was assigned a contribution score of 0 if either VH1 or VH4 was positive for the presence in either posture of reflux or obstruction in the IJV of interest.

The scoring scheme for the contribution of VH4 to the VHISS was the same as that for VH1, with the difference being that only blocks were considered. No points were assigned for segments and postures in which reflux had previously been detected under VH1.

The VH5 criterion had an overall VHISS contribution score between 0 and 4, calculated by assigning 0 to 2 points for each IJV. A -ΔCSA value was assigned a score of 2, whereas a ΔCSA value <7 mm^2^, corresponding to the 25th percentile of ΔCSA distribution in healthy controls, was assigned a score of 1. ΔCSA >7 mm^2 ^was assigned a score of 0.

The overall VHISS score was defined as a weighted sum of the scores contributed by each individual abnormal venous haemodynamics (A-VH) criterion. The formula for VHISS calculations was as follows:

$$\mathrm{VHISS}={\text{VHISS}}_{\text{1}}+{\text{VHISS}}_{\text{2}}+{\text{VHISS}}_{\text{3}}+{\text{VHISS}}_{\text{4}}+{\text{VHISS}}_{\text{5}}$$

The subscripts in this formula indicate the subscores for the five VH criteria.

### Statistical analysis

Statistical analysis was performed using the SPSS version 16.0 (SPSS, Inc., Chicago, IL, USA). The ages and proportions of females and males in the MS and HC groups were assessed with the Student's *t*-test and Fisher's exact test, respectively. The nonparametric Mann-Whitney *U *test was used to assess the VH differences between the MS and HC groups. Spearman correlation analysis was used to assess the relationship between PWI measures and the severity of CCSVI.

Since this was a preliminary exploratory study, we used a false discovery rate (FDR) correction [10] rather than a family-wise error rate (FWER) correction to correct for multiple comparisons on our outcome measures. FDR provides a statistical bound on the total percentage of incorrectly rejected null hypotheses rather than on the probability of any error occurring and is therefore considerably more powerful while still providing strong control. For the current work, we used an FDR threshold of 0.05, so we expect a 5% error rate for findings we consider significant. In the results, we report both uncorrected (*P*) and FDR-corrected (Q) statistics.

## Results

The demographic, clinical and VH characteristics of MS patients and HC groups are summarized in Table 1. The proportion of females to males (*P *= 0.67, Fisher's exact test) and the mean age of the two groups (*P *= 0.37) were similar. All MS patients were on disease-modifying therapy (seven were on subcutaneous interferon-β1a, two were on intramuscular interferon-β1a, four were on natalizumab and three were on glatiramer acetate).

**Table 1:** Demographic, clinical and venous hemodynamic characteristics of relapsing remitting MS patients and healthy controls^a^

Characteristic	MS patients (*n *= 16)	Healthy controls (*n *= 8)	*P *value
Female sex, *n *(%)	10 (63%)	6 (75%)	NS
Mean age, yr (±SD)	36.1 ± 7.3	33.1 ± 7.3	NS
Disease duration, mean (±SD)	7.5 ± 1.9		
Age at diagnosis, mean (±SD)	35.8 ± 9.2		
Expanded Disability Status Scale, mean (±SD) and median (range)	2.4 ± 0.9, 2.5 (1.0 to 3.5)		
Mean Multiple Sclerosis Functional Composite score (±SD)	-2.5 ± 0.03	-2.5 ± 0.02	NS
Mean treatment duration, yr (±SD)	4.3 ± 3.4		
Distribution of VH criteria, *n *(%)			
VH1	12 (75%)	0 (0%)	<0.001
VH2	14 (88%)	0 (0%)	
VH3	14 (88%)	1 (13%)	
VH4	13 (81%)	0 (0%)	
VH5	8 (50%)	0 (0%)	
Mean number of VH criteria (±SD)	3.8 ± 0.23	0.12 ± 0.35	<0.001
Mean VHISS (±SD)	8.9 ± 2.8	0 ± 0	<0.001

^a^MS, multiple sclerosis; VH, venous hemodynamic; SD, standard deviation; VHISS, venous hemodynamic insufficiency severity score; NS, nonsignificant. The differences between MS patients and healthy controls were assessed using the Student's *t*-test, Fisher's exact test and Mann-Whitney rank-sum test.

All 16 MS patients fulfilled the diagnosis of CCSVI (median VH = 4 and median VHISS = 9) and none of the HC (Table 1) (*P *< 0.001, Fisher's exact test). This means a CCSVI prevalence in this small group of MS patients of 100%, with prevalence of 0% in HC. The two venous scales, VHISS and number of VH criteria fulfilled, were significantly correlated with CCSVI diagnosis (*r *= 0.84 for VHISS, *r *= 0.84 for VH; *P *< 0.001 for both scales). Therefore, to decrease the number of comparisons, we used only VHISS for further analyses with PWI outcomes.

**Figure 3:**
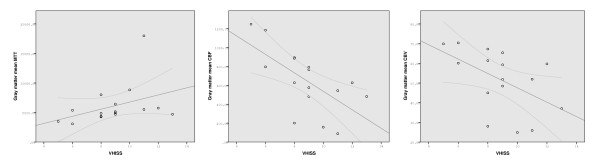
**Scatterplots showing the relationship between venous hemodynamic insufficiency severity score and gray matter mean transit time (left), cerebral blood flow (center) and cerebral blood volume (right) tissue perfusion parameters in patients with relapsing-remitting multiple sclerosis**.

**Figure 4:**
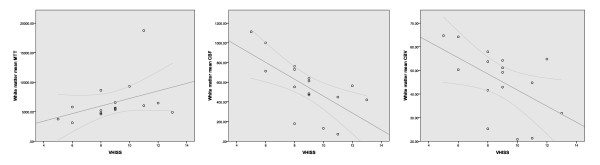
**Scatterplots showing the relationship between venous hemodynamic insufficiency severity score and white matter mean transit time (left), cerebral blood flow (center) and cerebral blood volume (right) tissue perfusion parameters in patients with relapsing-remitting multiple sclerosis**.

**Figure 5:**
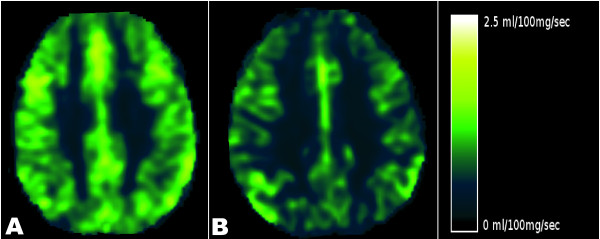
**Perfusion MRI study**. Left: Cerebral blood flow (CBF) in a 33-year-old, relapsing-remitting (RR) chronic cerebrospinal venous insufficiency (CCSVI) multiple sclerosis (MS) patient with a venous hemodynamic insufficiency severity score (VHISS) of 5. Right: CBF in a 38-year-old, RR CCSVI-MS patient with a VHISS of 12. The dark areas indicate lower CBF in the patient with higher VHISS.

There was a significant association between increased VHISS and decreased CBF in the majority of examined regions of the brain parenchyma in MS patients (Figures 3 to 5 and Table 2). The most robust correlations were observed for GM (Figure 3) and WM (Figure 4) (*r *= -0.70 to -0.71, *P *< 0.002, Q = 0.022) and for the putamen, thalamus, pulvinar nucleus of thalamus, globus pallidus and hippocampus (*r *= -0.59 to -0.71, *P *< 0.01, Q < 0.05). No results for correlation between VHISS and CBV or MTT survived multiple comparison correction (Figures 3 and 4 and Table 2). No significant relationship was observed between VHISS and PWI outcomes in HC.

**Table 2:** Spearman correlation coefficients, *P *values, and false discovery rate-corrected Q values between venous hemodynamic insufficiency severity score and perfusion-weighted measures in relapsing-remitting patients

	**Mean transit time**, *r*, *P*/Q	**Cerebral blood flow**, *r*, *P*/Q	**Cerebral blood volume**, *r*, *P*/Q
Gray matter	0.52,^a ^0.039/0.100^a^	-0.70,^b ^0.002/0.022^b^	-0.58,^a ^0.019/0.062^a^
White matter	0.53,^a ^0.034/0.094^a^	-0.71,^b ^0.002/0.022^b^	-0.49, 0.054/0.110
Caudate	0.50,^a ^0.049/0.110^a^	-0.38, 0.142/0.189	-0.30, 0.266/0.319
Putamen	0.47, 0.065/0.117	-0.72,^b ^0.002/0.022^b^	-0.42, 0.107/0.161
Globus pallidus	0.44, 0.087/0.142	-0.69,^b ^0.003/0.022^b^	-0.22, 0.404/0.428
Thalamus	0.46, 0.074/0.127	-0.65,^b ^0.007/0.042^b^	-0.62,^a ^0.011/0.052^a^
Pulvinar thalamus	0.25, 0.342/0.385	-0.67,^b ^0.003/0.022^b^	-0.50, 0.051/0.110
Hippocampus	0.34, 0.201/0.250	-0.61,^a ^0.012/0.052^a^	-0.41, 0.117/0.162
Amygdala	0.14, 0.604/0.604	-0.4, 0.091/0.142	-0.47, 0.064/0.117
Nucleus accumbens	0.49, 0.055/0.110	-0.60^a ^0.015/0.054^a^	-0.26, 0.336/0.384
Red nucleus	0.37, 0.163/0.210	-0.56,^b ^0.025/0.075^b^	-0.41, 0.112/0.161
Substantia nigra	0.17, 0.539/0.554	-0.60,^a ^0.013/0.052^a^	-0.23, 0.394/0.428

^a^Findings significant without false discovery rate (FDR) correction; ^b^significant correlations and findings surviving FDR correction.

## Discussion

This pilot study demonstrates that the presence and severity of CCSVI are associated with hypoperfusion of the brain parenchyma in patients with MS. In particular, a strong relationship was found between increased VHISS and decreased CBF in the GM, SGM and WM regions of the brain. No significant association was found in HC.

It has previously been demonstrated that MS patients show abnormal blood flow PWI patterns. These include increased MTT and decreased CBF and CBV within normal appearing WM and GM [4,11-15]. Perfusion abnormalities in the normal appearing WM are present from the earliest stages of the disease. The GM perfusion changes seem to appear somewhat later in the disease [4,13] and involve the thalamus, putamen and other SGM structures. PWI indices are also altered in both enhancing and nonenhancing lesions [12]. The severity of the perfusion changes is more pronounced in progressive MS compared to relapsing forms of the disease [11,13,15]. The hemodynamic abnormalities detected on PWI in patients with MS are currently interpreted as being a consequence of chronic inflammatory events related to local blood congestion and secondary hyperemia of the brain parenchyma [11,13,15]. Furthermore, at this time, it is not clear whether reduced perfusion of the WM and GM in MS patients is a sign of vascular pathology or decreased metabolic demand [5]. Alternatively, it can be hypothesized as the presence of a disorder that involves the major vasoactive substances. Norepinephrine, endothelin-1 and angiotensin II have been measured in their interaction with receptors only in the venous wall of the limbs, but not yet in CCSVI or in MS [16]. Although hypoperfusion is consistently present in MS lesions and GM and WM, the formation of new lesions is preceded by hyperperfusion changes [17]. Increased perfusion in the area of lesion formation could be a sign of vessel dilation mediated by proinflammatory cytokines.

An altered CBF pattern may be a consequence not only of local circulatory disturbances due to inflammatory mechanisms in acute or chronic phases, but instead could result from an outflow blockage situated far away from the lesions. CCSVI is a vascular condition described in MS patients that is characterized by stenoses caused by intraluminal defects such as web, septum, malformed valve or, rarely, by segmental hypoplasia/agenesis [1,2]. Stenosing lesions of CCSVI have been classified among the truncular venous malformation in a consensus document [18,19].

Therefore, CCSVI may impact local hemodynamics and overload microcirculation at places distant from the location of the mechanical stenosis, as in any condition of venous obstruction of the major trunks. Such a mechanism may lead to capillary hypertension and leakage, consistently contributing to inflammation [20]. In this pilot study, we have shown a strong relationship between the severity of CCSVI and hypoperfusion in the WM, GM and SGM. There are other examples of overload of the cerebral venous circulation, albeit triggered by different mechanisms from those of CCSVI, leading to hemodynamic abnormalities similar to those reported in this study. For example, the picture of dural arteriovenous fistula, characterized by plaques very similar to those of MS as well as by retrograde cortical venous drainage, shows abnormal perfusion parameters of CBV, CBF and MTT [21,22].

While we believe the present study provides important information about a poorly understood aspect of MS characteristics, it does have a number of limitations that motivate further work. First, the use of an FDR correction approach provides confidence in the overall body of results presented, but indicates that we expect approximately 5% of them to be incorrect. Thus, we can be confident that there is an overall relationship between VHISS and CBF in various areas of the brain, but need to further confirm individual regional findings in a more targeted study with a larger subject group. Second, when interpreting our perfusion results, it should be considered that absolute quantitation of CBF and CBV is challenging on dynamic contrast enhanced MRI. Some key parameters are estimated here and may not be correct. Nonetheless, the same MRI sequence, postprocessing steps and algorithm parameters were used for all subjects in all groups, so relative comparisons and correlations should still be reliable. Finally, several studies have reported that hypoperfusion of the brain parenchyma in MS patients advances with disease progression. It cannot be excluded that venous anomalies (CCSVI) may be secondary to reduced perfusion or that both are simply correlated with no direct causative relationship. Even if there were a causative relationship, the strongest *r *value we saw was 0.72, corresponding to a model explaining only about 50% of the overall variance seen. Thus, there may be other factors at work, and/or our measures may not be completely adequate to characterize individual hemodynamics. In either case, although we have established a relationship between CCSVI and reduced brain perfusion, the exact nature of that relationship remains uncertain and should be further investigated.

Several recent reports have presented evidence against the CCSVI hypothesis [23-25]. Particularly, a study of 56 MS patients and 20 HC found no differences in cerebrospinal venous drainage using transcranial and extracranial Doppler imaging. However, in this study, there were significant deviations from the original Doppler methodology adopted in previous and in the present studies [1,2,26]. The differences between the present study and other studies emphasize the need for a multimodal approach for the assessment of CCSVI.

Finally, accumulating evidence suggests that the increased energy demand of impulse conduction along excitable demyelinated axons and reduced axonal ATP production induce a chronic state of virtual hypoxia in demyelinated axons [27-29]. In response to such a state, further hypoperfusion of brain parenchima, facilitated by venous outflow disturbances, may contribute to chronic necrosis of axons and mitochondrial dysfunction.

## Conclusions

To the best of our knowledge, this pilot study is the first to report a significant relationship between the presence and severity of CCSVI and hypoperfusion in the brain parenchyma. These preliminary findings should be confirmed in a larger cohort of MS patients to ensure that they generalize to the MS population as a whole. Reduced perfusion could contribute to the known mechanisms of virtual hypoxia in degenerated axons.

## Abbreviations

CBF: cerebral blood flow; CBV: cerebral blood volume; CCSVI: chronic cerebrospinal venous insufficiency; IJV: internal jugular vein; MS: multiple sclerosis; MTT: mean transit time; VH: venous hemodynamics criteria; VHISS: Venous Hemodynamic Insufficiency Severity Score; VV: vertebral vein.

## Competing interests

PZ received funds for the present study from Hilarescere Foundation. BWG received personal compensation for consulting, speaking and serving on a scientific advisory board for Biogen Idec, Teva Neuroscience and EMD Serono. She also received financial support for research activities from the National Multiple Sclerosis Society (NMSS), the National Institutes of Health (NIH), Immune Tolerance Network (ITN), Teva Neuroscience, Biogen Idec, EMD Serono and Aspreva. FS received funds for the present study from Hilarescere Foundation. RZ received personal compensation from Teva Neuroscience, Biogen Idec, EMD Serono, Questcor and Genzyme for speaking and consulting. He also received financial support for research activities from the (NIH), the NMSS, the National Science Foundation, Biogen Idec, Teva Neuroscience, Genzyme, Bracco, Aspreva, Greatbatch and Jog for the Jake Foundation. All other authors have nothing to disclose.

## Authors' contributions

PZ and RZ equally contributed to conception and design, acquisition of data, analysis and interpretation of data; drafting the manuscript, revising it critically for important intellectual content and giving final approval of the version to be published. BWG and FS made substantial contributions to the study's conception and design and gave final approval of the version to be published. EM, MGD, CVS, AMM, DH, CK, EC, NB, CM and IB contributed to acquisition of data.

## Authors' information

PZ is Director of Vascular Diseases Center, University of Ferrara, Italy, and President of the International Society for Neurovascular Diseases. EM and AMM are both PhDs involved in the assessment of cerebral venous hemodynamics. RZ is the Director of the Buffalo Neuroimaging Analysis Center, University at Buffalo, Buffalo, NY, USA, and Associate Professor of Neurology. MGD, CVS, CK, EC, NB and CM are researchers at the Buffalo Neuroimaging Analysis Center, University at Buffalo, Buffalo, NY, USA.

BWG is Associate Professor and DH is a neurologist at the Jacobs Neurological Institute, University at Buffalo, Buffalo, NY, USA. FS is Head of the MS Center and IBPhD student at Bellaria Neurosciences, Bologna, Italy.

## Pre-publication history

The pre-publication history for this paper can be accessed here:

http://www.biomedcentral.com/1741-7015/9/22/prepub
